# *Ganoderma lucidum*-derived polysaccharide enhances coix oil-based microemulsion on stability and lung cancer-targeted therapy

**DOI:** 10.1080/10717544.2018.1516006

**Published:** 2018-10-20

**Authors:** Jian Guo, Chengtian Yuan, Mengmeng Huang, Yuping Liu, Yunyan Chen, Congyan Liu, Yan Chen

**Affiliations:** a Affiliated Hospital of Integrated Traditional Chinese and Western Medicine, Nanjing University of Chinese Medicine, Nanjing, China;; b Jiangsu Province Academy of Traditional Chinese Medicine, Nanjing, China

**Keywords:** *Ganoderma lucidum* polysaccharides, anti-lung cancer therapy, multicomponent microemulsion, fluorescence resonance energy transfer, spatial structure characterization

## Abstract

The aim of this study is to explore the influence of *Ganoderma lucidum*-derived polysaccharides (GLP) to coix oil-based microemulsion on pharmaceutical performance and anti-lung cancer treatment. GLP-integrated coix oil-based microemulsion (MEs(PS-GLP)) exhibited a clear spherical shape, small particle size, and good hydrodynamics similar to the coix oil-based microemulsion, but showed a lower zeta potential and a better stability. Fluorescence resonance energy transfer analysis presented that GLP was integrated with microemulsion as a single system. Notably, the average molecular distance between polysaccharide and microemulsion was approximately 1.7 nm. The half-maximal inhibitory concentration of MEs(PS-GLP) against A549 cells was about 119 μg/mL. *In vivo* imaging studies showed that introduction of GLP promoted the tumor-specific accumulation of microemulsion in comparison with controls. *In vivo,* antitumor results showed that MEs(PS-GLP) markedly inhibited the tumor growth of A549-bearing xenograft nude mice and obviously improve the serum immune index. Collectively, this study demonstrates the potential mechanism of spatial relation between polysaccharides and microemulsion and validates the significances of GLP on tumoral accumulation and antitumor efficacy.

## 1. Introduction

Polysaccharides are polymeric carbohydrate macromolecules that play important roles in the growth and development of living organisms. Pharmacological studies have confirmed that can participate in the physiological metabolism of the body and have biological activities such as immunomodulatory, anti-oxidant, anti-diabetic, antibiotic, anti-inflammatory, and anti-tumor activities (Xie et al., 2016; Yu et al., 2018). The use of polysaccharides as bioactive components and medicinal products can be found throughout the world, particularly in the traditional Chinese medicines. *Ganoderma lucidum* is a widely used traditional Chinese fungus medicine for centuries to prevent and treatment of human diseases (Qu et al., 2014). *Ganoderma lucidum* polysaccharides (GLP) are one type of main bioactive components in *Ganoderma lucidum* and they exhibit various significant pharmacological activities such as immunomodulation, anti-obesity, anti-oxidant activities, and anti-tumor activities (Xu et al., 2011; Shi et al., 2013; Jiang et al., 2015; Jin et al., 2016; Chang et al., 2015). For example, Chang et al. showed that high molecular weight polysaccharides (4300 kDa) isolated from the *Ganoderma lucidum* mycelium extract produce anti-obesity and microbiota-modulating effects in mice fed a high-fat diet (Chang et al., 2015). Shen et al. revealed that the GLP can suppress HepG2 cells via the regulation of the hepatic miRNAs (miR-10b, miR-23a, miR-92a, and miR-199a-3p) (Shen et al., 2014). However, the GLP also have some drawbacks such as difficulty in purification, low oral absorption, large-dose-dependent and brief biological half-life, which limit its development into a commercial pharmaceutical formulation for clinical application (Liu et al., 2016b).

Coix as a traditional herbal has been used widely for more than one thousand years. Benefiting from modern supercritical extraction technology, coix seed oil extracted from coix has a promising potential in anticancer treatment due to the unique mechanism (Lee et al., 2008). Coix seed oil has been developed as an anticancer drug called Kanglaite^®^ injection, which was applied clinically in China and Russia (Wang et al., 2017). However, water insolubility and low oral bioavailability limited the improvement on anticancer efficacy. Thus, it is necessary to develop a drug delivery system, which can improve oral absorption and cancer treatment.

The spatial conformation of natural polysaccharides determines water solubility and bioadhesion, which can be applied in the fields of biomaterials or pharmaceutical formulation (Yang et al., 2014; Kang et al., 2015; Yang et al., 2015; Li et al., 2017b). In previous studies, we had developed the coixan-integrated microemulsions to improve oral absorption of coixan and anti-tumor efficacy of coix seed oil (Liu et al., 2016a; Qu et al., 2017b). We previously reported that coix seed oil was capable of acting as oil excipient in the microemulsion delivery system. Likewise, introduction of polysaccharide could reduce the amount of surfactant in formation of microemulsion, leading to an improvement of drug loading efficiency and anticancer efficacy (Liu et al., 2016a; Li et al., 2017b). However, the pathway of interaction between polysaccharide and microemulsion is still unclear.

In this study, we focused on the changes in pharmaceutics characterization and tumor targeting after integration of GLP into microemulsion. Furthermore, we investigated the interaction mechanism of particle and GLP using fluorescence resonance energy transfer (FRET) technology. In addition, the stability, *in vitro* and *in vivo* antitumor effect of microemulsions with and without GLP were also evaluated.

## 2. Materials and methods

### 2.1. Materials

Coix seed oil was obtained by supercritical CO_2_ extraction technology (purity >85%, determined by ultraviolet spectroscopy). Cremophor^®^RH40 was purchased from BASF.SE (Germany) and polyethylene glycol (PEG) 400 was purchased from Sigma-Aldrich Co., Ltd (Sigma-Aldrich, St. Louis, MO, USA). Labrafil^®^M 1944CS was received as a gift from Gattefossé Co., Ltd (Nanterre Cedex, France). As described previously, *Ganoderma lucidum* polysaccharide was prepared by water extraction and purified by alcohol precipitation by our group (Zhu et al., 2006, Zhu et al., 2012; Wang et al., 2018). Chitosan (MW 5 kDa) was purchased from Jinan Haidebei Marine Bioengineering Co., Ltd (Jinan, China). Dextran (MW 70 kDa) was purchased from Klamar reagent (Shanghai, China). Sephadex-G150 used for gel sieving was purchased from Pharmacia Limited (Sweden). Rh123 kit (Rh123 solution with concentration of 1 mg/mL) was purchased from KeyGen BioTECH (Nanjing, China). Roswell Park Memorial Institute 1640 medium, Dulbecco’s modified Eagle medium, fetal bovine serum, penicillin-streptomycin solution, and phosphate-buffered saline were purchased from Thermo Fisher Scientific Inc. (Waltham, MA, USA). The MTT [3-(4,5-dimethylthiazol-2-yl)-2,5-diphenyltetrazolium bromide] was sourced from Amresco (Solon, OH, USA). Fluorescein 5-isothiocyanate (FITC) was supplied by Sigma-Aldrich Co., Ltd (Sigma-Aldrich, St. Louis, MO, USA). All other reagents were analytical reagent grade.

### 2.2. Preparation of microemulsions

Pseudoternary phase diagram is a useful tool for determination of the zone of microemulsion formation. Thus, we use the pseudoternary phase diagram by the aqueous titration method as described in our previous reports to screen out the optimal formulation of microemulsion (Qu et al., 2017a). In this study, coix seed oil was used as the oil phase, while Cremophor RH40, Tween-80, OP-10, and Tween-20 were selected as candidates for the surfactant. PEG400, ethanol, and isopropanol were used to screen for the optimal cosurfactant. And the mass ratio (K_m_) of surfactant and cosurfactant was settled as 1:1, 2:1, and 3:1. The critical point of blank microemulsion (MEs(PS-Free)) formation was evaluated. To explore the replacement of surfactant of GLP, the part substitution of GLP for equivalent surfactant was used for the preparation of microemulsion and the size was also determined. The GLP-integrated microemulsion (MEs(PS-GLP)) was prepared. As control, microemulsion using Labrafil^®^ M 1944CS as the oil phase (MEs(1944CS)) was prepared as well (Su et al., 2017).

### 2.3. Characterization of microemulsions

The particle size and zeta potential of different microemulsion formulations were measured by dynamic light scattering (DLS, Nano ZS, Malvern Instruments Ltd, Malvern, UK). The morphology of the microemulsion was evaluated by transmission electron microscopy (TEM, Tecnai 12, Philips, Amsterdam, Netherlands). Each sample was prepared by dropping 15 μL of microemulsion on a holey carbon-coated copper and dried prior to measurement. To characterize the phase inversion temperature of MEs(PS-Free) and MEs(PS-GLP), the conductivity of the continuous phase was measured with a voltmeter (Jenway 4071, U.K.).

### 2.4. Stability study

The stability of microemulsions was investigated under the various critical factors including freeze-drying, centrifugation, dilution and cool-heat cycle. Briefly, the microemulsions were resuspended in deionized water after freeze-drying, diluted 10, 50, 100, 500, and 1000-fold with deionized water, centrifuged at 13,000 rpm for 5 min, stored at 4 °C, and 24 °C in 7 days under cool-heat cycle, to observe any morphologic and size changes.

### 2.5. Mechanism studies of the interaction between microemulsions and polysaccharides

#### 2.5.1. Gel-permeation chromatograph analysis

In this study, a gel permeation chromatography (8 g Sephadex-G150, 100 mm ×40 mm) was used to determine whether the polysaccharides and microemulsions were combined together. 1 ml of FITC-labeled chitosan (CSF), MEs(PS-Free) and their mixture were injected separately, each sample was eluted with the ultrapure water, and the eluent was collected from 2 mL to 40 mL, then the UV-VIS absorption at 492 nm of each eluent was determined. The FITC-labeled dextran (DTF) and its loaded microemulsions were performed as the same method. The FITC-labeled dextran (DTF) and its loaded microemulsions were performed as the same method.

#### 2.5.2. Fluorescence resonance energy transfer analysis

FRET analysis was performed to investigate the interactions of polysaccharides and microemulsions. In the experiment, Rh123 and FITC were used as FRET pairs. The Rh123-loaded microemulsion (Rh123 MEs) was prepared and was the acceptor (Ex = 521 nm, Em = 538 nm), whereas the FITC-labeled polysaccharides were the donor (CSF: Ex = 494 nm, Em = 517 nm; DTF: Ex = 516 nm, Em = 521 nm). For CSF-Rh123 MEs, the CSF solution and Rh123 MEs were mixed and incubated for 30 min at room temperature. Fluorescence spectra were recorded using a Hitachi F7000 fluorospectro photometer.

Take FITC-labeled dextran (DTF)-Rh123 MEs as an example, the distance between the DTF and Rh123 MEs is the molecular separation (R) of donor and acceptor, and can be calculated by Equation (1) (Selvin, 2000):

(1) $$E=1-\frac{\tau_{DA}}{\tau_{D}}=\frac{R_{0}^{6}}{R_{0}^{6}+R^{6}}$$

where E is FRET efficiency, R_0_ is the Förster radius, R is the molecular separation, $\tau_{DA}$ is the lifetime of donor when acceptor exist, and $\tau_{D}$ is the lifetime of the donor in the absence of acceptor. When E, R_0_, $\tau_{DA}$ and $\tau_{D}$ were known, R can be calculated. The Förster radius R_0_ can be given by the Equation (2) and Equation (3) (Peter et al., 2005):

(2) $$R_{0}=0.0211（K^{2}QJn^{-4}{）}^{1/6}$$

(3) $$J=\frac{\int_{0}^{\infty }F_{D}(\lambda )\varepsilon_{A}(\lambda )\lambda^{4}d\lambda }{\int_{0}^{\infty }F_{D}(\lambda )d\lambda }$$

Where *n* is the refractive index, Q is the fluorescence quantum yield of donor, K is a parameter being related to the orientation of donor and acceptor. J is spectral overlap integral, $F_{D}(\lambda )$ is the corrected fluorescence intensity of the donor, $\varepsilon_{A}(\lambda )$ is the extinction coefficient of acceptor.

The fluorescence spectra of the donor and acceptor were recorded on Hitachi F7000 fluorospectro photometer, while the fluorescence lifetime was collected on HORIBA FL3-TCSPC Transient State Fluorescence Spectrometer.

### 2.6. Cell culture and animals

A human lung cancer A549 cell line and a human colon adenocarcinoma Caco-2 cell line were supplied by the Cell Bank at the Chinese Academy of Sciences. The A549 cells were cultured in Roswell Park Memorial Institute-1640 medium with 10% (v/v) fetal bovine serum, the Caco-2 cells were grown in Dulbecco’s modified Eagle medium with 10% (v/v) fetal bovine serum, 100 U/mL penicillin and 100 µg/mL streptomycin were added to the medium additionally, cells were cultured in an incubator at 37 °C under an atmosphere of 5% CO_2_ and 90% relative humidity. The cells were passaged by trypsin at a split ratio of 1:8 every 4–5 days (at 80% confluence).

Athymic nude BALB/c mice (18–20 g) were supplied by SiLaike laboratory animal Co. Ltd (Shanghai, China), which were received care in accordance with the Guide for Care and Use of Laboratory Animals, approved by the Animal Experimentation Ethics Committee of Jiangsu Provincial Academy of Traditional Chinese Medicine. Animals were maintained under a group of five with free access to food and water and a 12 h light/dark cycle. All animals acclimated to the animal facility for at least 7 days before experimentation. All possible parameters that may cause social stress, like group size, type (treated and nontreated), etc., among the experimental animals, were carefully monitored and avoided. Animals were observed daily for any behavioral abnormalities and weighed weekly.

### 2.7. Cytotoxicity studies

To evaluate the potential toxicity of MEs(PS-GLP) to tumor cells, the antiproliferative effects against A549 cells were assessed using the MTT assay. In brief, A549 cells were seeded at a density of 5 × 10^4^ cells/well in 96-well plate. Afterwards, the culture medium was replaced by 200 µL of the test solutions and incubated for an additional 24 hours at 37 °C. Next, 20 µL of 5 mg/mL MTT solution was added to each well for another 4 hours. The medium was removed, and 150 µL of DMSO was added to each well to dissolve the formazan crystals. The optical density of each well at 570 nm was recorded by a microplate reader (Thermo Scientific, Waltham, MA, USA). The relative cell viability rate was calculated as follows: viability (%)=absorbance of sample/absorbance of control ×100%. The half-maximal inhibitory concentration (IC_50_) values were calculated using GraphPad Prism software. The intestinal epithelial toxicity of MEs(1944MS), MEs (PS-Free), and MEs(PS-GLP) was also investigated on Caco-2 cells using the same method.

### 2.8. *In vivo* distribution analysis

For *in vivo* imaging analysis, Cy5-labeled MEs(PS-GLP) was prepared to track the microemulsions. When the tumor reached around 60 mm^3^, the mice (*n* = 3) were intragastrically administrated with Cy5-labeled MEs(PS-GLP) at Cy5 dose of 1.2 mg/kg. Images of the isoflurane-anaesthetized mice were taken at 1, 2, 4, 6, 8, and 24 h postadministration using the IVIS Lumina imaging system. The fluorescent images were taken at the predetermined time points. At 48 h after administration, the mice were euthanized. Other Cy5 formulations, including the free Cy5 solution and MEs(PS-Free) were taken as references.

### 2.9. *In vivo* anti-tumor effect evaluation

The *in vivo* antitumor efficacy of MEs(PS-GLP) was evaluated on the lung cancer models. When the tumor reached around 80 mm^3^, the A549-bearing mice were intragastrically administrated with MEs(PS-Free), MEs(PS-GLP) and MEs-GLP mixture at coix seed oil dosage of 2.5 g/kg once daily for 14 days. Saline and Kanglaite^®^ injection were taken as references. Tumor size and body weight of each mouse were measured every other day. Tumor growth was monitored by measuring the perpendicular diameter of the tumor with calipers. The estimated volume was calculated according to the formula: tumor volume (cm^3^) = 0.5 × length × width^2^. At day 28, after 7 days of the therapeutic endpoint, the mice were sacrificed and tumor tissues were extracted to record weight. The liver and spleen were collected and weighed to evaluated safety. The liver and spleen indices were calculated as W/W_0_, where W and W_0_ are the liver or spleen weight and body weight after treatment, respectively.

### 2.10. Data analysis

All data are expressed as mean ± standard deviation (SD). Statistical analysis was performed by Graphpad Prism software using two-tailed Student’s *t*-test. The differences are assumed to be statistically significant at *p < .05 and **p < .01.

## 3. Results and discussion

### 3.1. Preparation and characterization of MEs(PS-GLP)

The microemulsion was prepared by aqueous titration method and pseudoternary phase diagrams were constructed to identify the optimal microemulsion formulation by comparison of the area of the microemulsion (Zhao et al., 2018). As depicted in Figure S1, the total area of the microemulsion reached the maximum when Cremophor RH40 and PEG400 were used as the surfactant and cosurfactant respectively with the K_m_ at 3:1. Thereby, the weight ratio of coix seed oil, RH40 and PEG400 were determined as 400/200/67 in preparation of microemulsion. As shown in Figure S2(a), the particle size of microemulsion significantly increased with the gradual decrease in the amount of RH40. The size of particle with the weight ratio of coix oil to RH40 at 400/200 was obviously larger than that of ratio at 400/175 (*p* < .01), but remarkably smaller than that of ratio at 400/225 (*p* < .05). In addition, various sizes influenced the clarity of microemulsion solution that probably determined the *in vitro* stability and solubilization. Therefore, the weight ratio of coix oil to RH40 at 400/200 was considered as the critical point of microemulsion formation.

**Figure 1.**
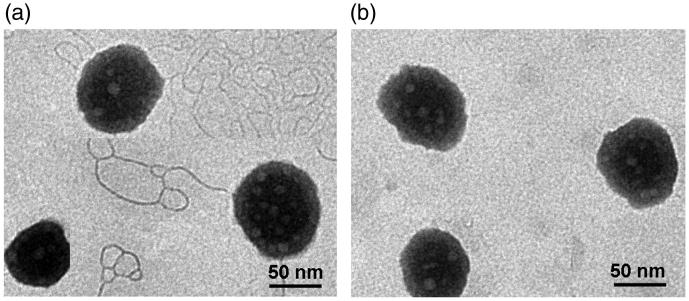
Morphology of (a) MEs(PS-Free) and (b) MEs(PS-GLP).

To validate the feasibility of GLP as a surfactant, we replaced RH40 with equivalent GLP in the preparation of MEs(PS-GLP). GLP was prepared by classical water extraction and alcohol precipitation method, and dialyzed by a 14,000 kD dialysis membrane in double distilled water for 24 h to eliminate small molecules interfering (Zhu et al., 2006, Zhu et al., 2012; Wang et al., 2018). Furthermore, the experiment found that GLP has no obvious absorption peak at 260 in ultraviolet spectrum, suggesting no detectable nucleic acid existed in GLP. As shown in Figure S2(b), the size of MEs (PS-GLP) was smaller than 100 nm with a transparent appearance when the weight ratio of GLP (wt%) was lower than 7.50%. Notably, with further addition of GLP, the microemulsions became opaque with a sharp increase in size. Therefore, the added wt% of GLP was 7.50% in the following studies. The weight ratio of various components in formulation of MEs(PS-GLP) was as follows: 400/150/67/50, coix seed oil/RH4 PEG400/GLP. The obtained MEs(PS-GLP) had an average particle size of 87.94 nm, PDI of 0.092 and a zeta potential of −12.10 mV (Table 1). In this study, the phase inversion temperatures of MEs(PS-GLP) and MEs(PS-Free) were detected (Table 1). Phase inversion temperature was slightly decreased to 85 °C in the microemulsions integrated with GLP. The results indicated that the presence of a carbohydrate or in some cases a drug, particularly hydrophilic in nature as GLP, can act to lower the phase inversion temperature. The morphology of the microemulsions was investigated by TEM (Figure 1). The MEs(PS-Free) was spherical droplet with smooth surface, after GLP integrated, the morphology of MEs(PS-GLP) still maintained a spherical structure with regular surface, which was consistent with the results measured by DLS.

**Table 1.** Characterization of MEs(PS-Free) and MEs(PS-GLP). All the data are presented as mean ± SD (*n* = 3).

Formulations	Size (nm)	PDI	Zeta (mV)	Phase inversion temperature (°C)
MEs(PS-Free)	86.67 ± 3.36	0.089 ± 0.021	−14.50 ± 1.01	95
MEs(PS-GLP)	87.94 ± 3.17	0.092 ± 0.033	−12.10 ± 0.70	85

### 3.2. Stability of microemulsion

In order to investigate the changes in stability of microemulsion after introduction of GLP, the size of MEs(PS-GLP) and MEs(PS-Free) was recorded using various intervention methods, including freeze-drying, centrifugation, dilution and cool-heat cycle. As shown in Figure S3(a), both of two kinds of microemulsion had stable size range around 80 nm, representing that integration of GLP did not influence the redissolution of freeze-dried power of microemulsion. In addition, we did not observe significant change of size, flocculation or precipitate after centrifugation at 13,000 rpm for 5 min (Figure S3b). After different dilution times ranged from 10 to 1000-fold, the size of microemulsions maintained about 80 nm (Figure S3c), indicating a potent stability *in vivo*. The thermostability was evaluated for the microemulsions by the cool-heat cycle of 4 °C and 37 °C. The temperature changes every 12 hours and the cycle operate 7 days. There was little change in size, and no obvious turbidity or flocculation was observed (Figure S3d), indicating the microemulsions had an acceptable thermostability. Taken together, MEs(PS-GLP) had a good stability similar to MEs(PS-GLP).

### 3.3. Mechanism of interaction between microemulsions and polysaccharides

#### 3.3.1. Charge attraction analysis

**Figure 2.**
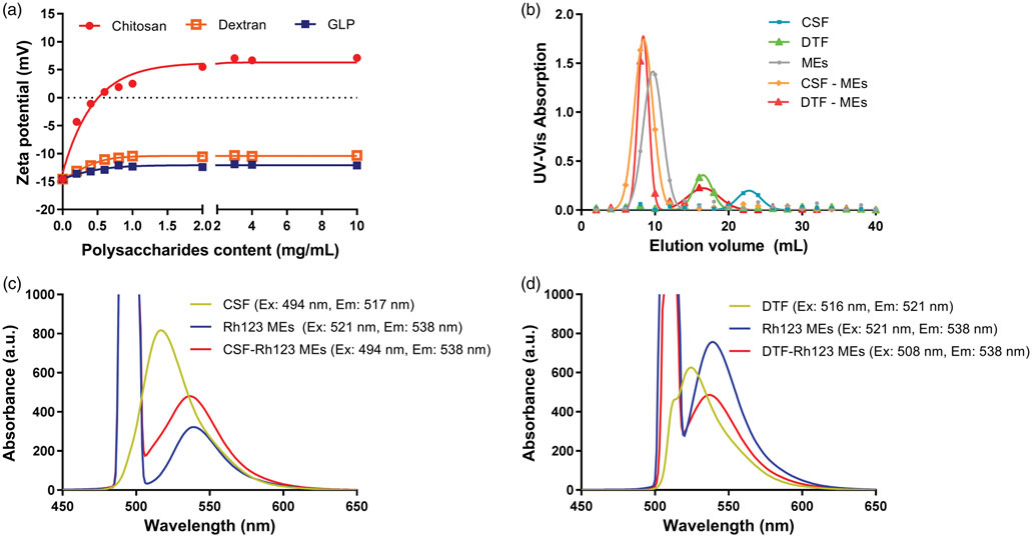
(a) Zeta potential of microemulsions coated by chitosan, dextran, and GLP with concentration of 0–10 mg/mL. (b) UV-Vis absorption of CSF, DTF, blank microemulsions and microemulsions coated by CSF and DTF (CSF-MEs, DTF-MEs). FRET analysis of fluorescence emission spectra of (c) CSF, Rh123 MEs and CSF-Rh123 MEs, (d) DTF, Rh123 MEs and DTF-Rh123 MEs.

Previous studies had shown that nano preparations with negative charge like microemulsions could be coated by chitosan through electrostatic adsorption (Shah et al., 2016; Li et al., 2017a). In this study, chitosan was taken as positive control, and the zeta potential of different polysaccharides integrated microemulsions were also measured. As shown in Figure 2(a), the zeta potential of MEs(PS-Free) was found to be negative, agreeing with the result above. After the addition of positively charged chitosan, the zeta potential of microemulsions rose up to be positive and reached peak potential of <7 mV due to the electrostatic attraction. On the contrary, the zeta potential was not reversed after the addition of neutral charged dextran. Moreover, with the addition of GLP, the zeta potential of microemulsions was increased but only reached the peak of approximately −12 mV, which still remained to be negative. These results suggested that the GLP can be successfully coated onto the surface of microemulsion, and the interaction between GLP and microemulsions was not the electrostatic attraction as zeta potential maintained negative after GLP addition.

The combination of GLP-microemulsions was further investigated through gel permeation chromatograph experiment. Gel permeation chromatograph is a type of size exclusion chromatography that separates analytes on the basis of size (Du et al., 2017). It is a useful analytical tool for monitoring the molecular weight and distribution of polymers. In order to eliminate the interference of impurities in GLP and make the results more suitable for other polysaccharides extracted from traditional Chinese medicine, dextran were used as substitution of GLP owing to the similarity of monosaccharide composition and glycosidic bonds. Therefore, the DTF was synthesized and identified by FT-IR and ^1 ^H NMR spectra (Figure S4). The CSF was synthesized for positive control. The final replacement degree of FITC to CSF and DTF was 6.25% and 7.02%, respectively. The UV-Vis absorption of CSF, DTF, and microemulison was detected in gel permeation chromatograph analysis (Figure S5). According to the results, the wavelength of 492 nm was finally chosen to be suitable for detection. The passage of larger molecular weight compound through the gel column takes a short time and has a small elution volume. As shown in Figure 2(b), the blank microemulsion of MEs equilibrated by ultrapure water showed one peak at 10 mL with UV-Vis absorbance of 1.384 using Sephadex G-150 gel filtration, while CSF solution showed one peak at 22 mL with the absorbance of 0.172. After incubated with CSF for 0.5 h, the peak of CSF-MEs was moved to 8 mL with UV-Vis absorbance of 1.634, and the absorption of CSF was almost eliminated, which indicated the improvement of MEs molecular weight due to the integration of CSF into the MEs. Similar result was observed as with the DTF solution. The DTF solution absorption peak shown in 16 mL with a value of 0.330 and the peak of DTF-MEs moved to 8 mL after incubated with DTF for 0.5 h. In addition, the absorbance of DTF-MEs was improved to 1.520 at the volume of 8 mL, while the DTF was reduced to 0.228. These results verified the successful combination of DTF with MEs.

#### 3.3.2. Fluorescence resonance energy transfer analysis

In order to validate the integration of MEs (PS-GLP), FRET technology was used to figure out the potential distance between polysaccharide and microemulsion. Here, chitosan and dextran were both labeled with FITC as CSF and DTF, respectively, and microemulsion was modified with Rh123 as well. As displayed in Figure S6, CSF has a maximum excitation wavelength at 494 nm and a maximum emission wavelength at 517 nm, which is similar to free FITC. Likewise, DTF solution showed an extinction peak at 516 nm with an emission peak at 521 nm. Typically, Rh123 MEs exhibited an extinction maximum at 521 nm and an emission maximum at 538 nm, indicating a FRET acceptor to donator (CSF or DTF).

As shown in Figure 2(c), CSF-Rh123 MEs existed an obvious shift of emission wavelength compared with CSF solution alone after excitation at 494 nm, indicating that the distance between CSF and Rh123 MEs was close enough (<10 nm). It could be explained by charge attraction of positive chitosan and negative microemulsion. As the control, the physical mixture of CSF and Rh123 did not occurred the similar phenomenon mentioned above (Figure S7a). In view of these results, we infer that CSF is probably coated on the surface of microemulsions. In contrast, DTF is a neutral polysaccharide that has little possibility of linking with microemulsion through charge attraction. Since the maximum excitation wavelength (517.4 nm) and emission wavelength of DTF (521 nm) are close to each other, the excitation wavelength in the FRET experiment was chosen 508 nm to reduce the interference of excitation light source on emission spectrum of DTF. As shown in Figure 2(d), the DTF-Rh123 MEs and Rh123 MEs showed maximum emission at 538 nm, along with a 17 nm Stokes shift compared with DTF solution. It meant that the photon, produced by DTF molecule after irradiation of light, was absorbed by Rh123 MEs and translated to new photon with wavelength of 538 nm. It is well known that the FRET would happen if only the distance between the donor and the acceptor was less than 10 nm. These findings provide the decisive evidence that DTF and microemulsion integrated into a holistic system. By contrast, the physical mixture of DTF and Rh123 solution showed an emission peak at approximately 523 nm (Figure S7b). The above results confirm that DTF and microemulsion are tightly combined into one system, but not as electrostatically attracted as CSF.

The DTF was used to replace GLP to quantify the interaction between the GLP-microemulsions. The distance between DTF and Rh123 MEs was calculated according to the formula in section 2.5. The fluorescence emission spectrum of the donor of DTF and the excitation spectrum of the acceptor of Rh123 MEs were described in Figure S8(a) and S8(b), and according to the Equation (3), the J was calculated as 1.323 × 10^18^. The donor and acceptor are randomly arranged, so K^2^ = 2/3. The refractive index of water was 1.333 and Q_DTF_ was 0.02. The fluorescence lifetimes were collected in Figure S8(c). $\tau_{DA}$ was 2.59 ns and $\tau_{D}$ was 3.83 ns. Finally, according to the Equation (1) and Equation (2), the distance between DTF and Rh123 MEs was calculated to be 1.7 nm. From the above results, it can be concluded that the GLP was inserted into the spherically structure of the microemulsions.

### 3.4. Cytotoxicity evaluation

**Figure 3.**
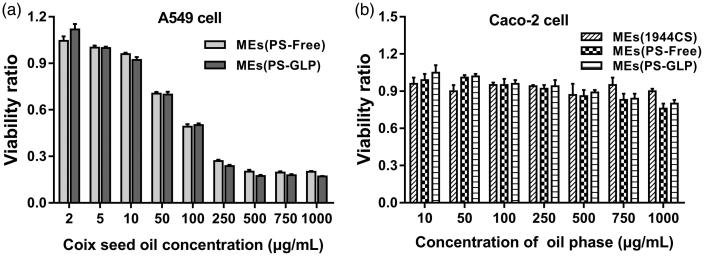
(a) Cell viabilities of A549 cells incubated with MEs (PS-free) and MEs (PS-GLP) at various coix oil concentrations for 24 h. (b) Cell viabilities of Caco-2 cells incubated with MEs (1944CS), MEs (PS-Free) and MEs (PS-GLP) at various oil phase concentrations for 24 h. All the data are presented as mean ± SD (*n* = 6).

Coix seed oil is the main active constituent in Kanglaite^®^ injection approved for the treatment of non-small cell lung cancer and advanced hepatocellular carcinoma by the China Food and Drug Administration in 1995 (Schwartzberg et al., 2017; Wang et al., 2017; Yang et al., 2018). Although GLP has not been a commercial formulation yet, the immunologic regulation to cancer cells was proved by previous reports (Zeng et al., 2018). However, the antiproliferative effect of MEs(PS-GLP) and GLP solution against A549 cells has not studied till now. As exhibited in Figure 3(a), both MEs(PS-Free) and MEs(PS-GLP) exhibited a dose-dependent cytotoxicity. The IC_50_ of MEs(PS-Free) and MEs(PS-GLP) were calculated to be 127 μg coix seed oil/mL and 119 μg coix seed oil/mL, respectively. There is no significant difference between the two kinds of microemulsions. Besides, GLP had no obvious cytotoxicity against A549 cells at the concentrations ranged from 2–1000 μg/mL. It suggested that introduction of GLP into microemulsion did not reduce its antiproliferative effect.

As an oral drug delivery system, the intestinal toxicity is an important consideration factor. Caco-2 cell is widely used to mimic the intestinal epithelial cells for the similar structure and function, as well as the relevant enzymes. As shown in Figure 3(b) and S9, the cytotoxicity of various microemulsions against intestinal epithelial cell was evaluated by incubation with Caco-2 cells for 24 h and 48 h, respectively. The blank MEs containing the same amount of RH40 and PEG400 was prepared as negative control. The blank MEs showed no cytotoxicity against Caco-2 cells at all predetermined concentrations. MEs(PS-GLP) showed an enhancement on cell viability related to MEs(PS-Free). The viability ratio of Caco-2 cells treated with MEs(PS-GLP) was approximately 0.8 even the coix seed oil concentration increased upto 1000 μg/mL. It revealed that the MEs(PS-GLP) had an acceptable safety in the intestinal tract during oral administration.

### 3.5. Biodistribution

**Figure 4.**
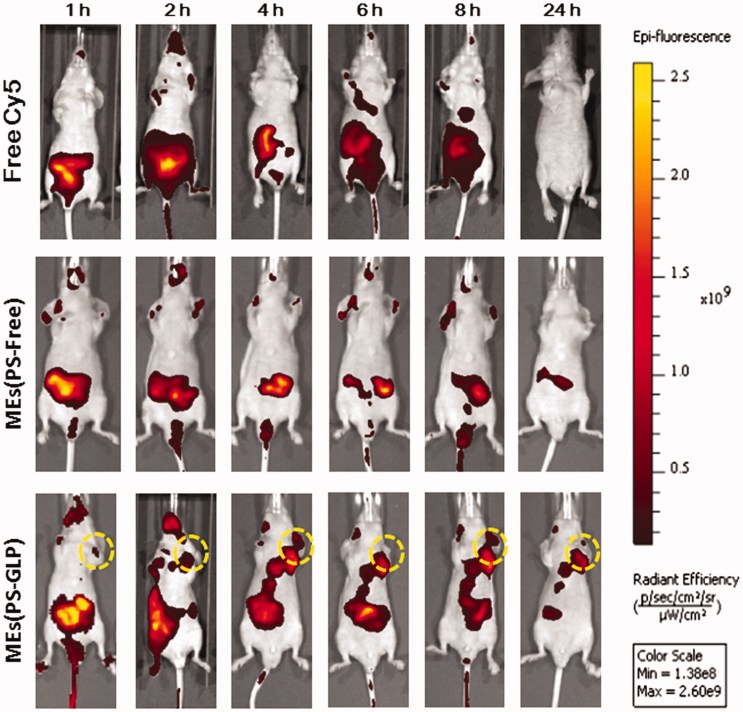
*In vivo* images after intragastric administration of Cy5 solution, Cy5-labeled MEs (PS-free) and Cy5-labeled MEs (PS-GLP) in A549 tumor-bearing mice.

According to our previous studies, polysaccharide-integrated microemulsion exhibited an improvement in tumor accumulation after oral administration, probably related to the enhanced passive tumor targeting (Qu et al., 2017c). In this study, the biodistribution of MEs(PS-GLP)-labeled with Cy-5 was investigated using A549 xenograft nude mice as animal model by near-infrared imaging system. As shown in Figure 4, free Cy5 mainly distributed in the intestinal tract and eliminated rapidly from body within 24 h post-intragastric administration. At 8 h post administration, the Cy5 fluorescence signal was still observed in the intestine of the nude mice treated with MEs(PS-GLP). However, almost no fluorescence signal was detected in the MEs(PS-Free) group. It is suggested that the integration of polysaccharides could prolong the intestinal retention of microemulsion, probably attributed to the improvement of intestinal adhesion offered by polysaccharides (Wang et al., 2016). More importantly, near-infrared signal of MEs(PS-GLP) at tumor tissue was obviously visualized from 2 to 24 h after administration in MEs(PS-GLP) group that was not observed in MEs(PS-Free) group, exhibiting the advantage in tumor accumulation of MEs(PS-GLP).

### 3.6. Antitumor efficiency *in vivo*

**Figure 5.**
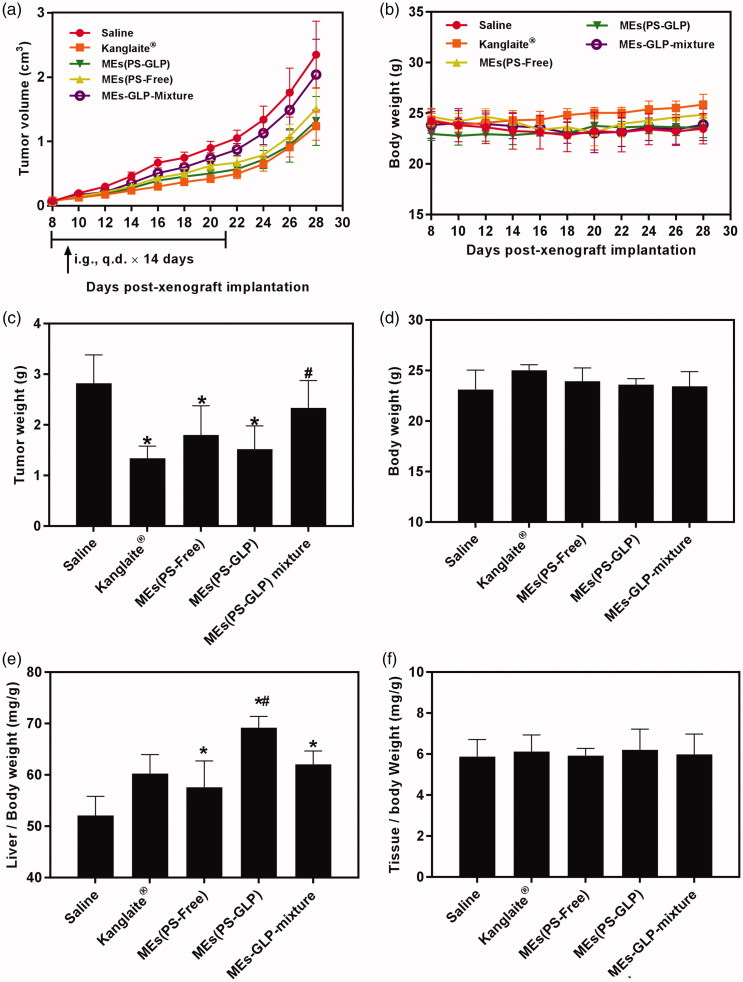
Evaluation of antitumor efficiency *in vivo*. The curve of (a) tumor growth and (b) body weight change of A549 tumor-bearing mice after various treatments for three weeks. The (c) tumor weight and (d) body weight of A549 tumor-bearing mice after various treatments on Day 30. *p < 0.05, compared with saline group. #*p* < 0.05 compared with Kanglaite^®^ injection group. The (e) liver index and (f) spleen index of A549 tumor-bearing mice after various treatments in Day 30. *p < 0.05, compared with saline group. #*p* < 0.05 compared with MEs-GLP mixture group. All the data are presented as mean ± SD (*n* = 8).

To validate the advantages of integrating GLP into coix seed oil microemulsion system, the antitumor efficacy was performed through intragastrical administration of MEs(PS-GLP) on the A549 tumor-bearing mice. As shown in Figure 5(a), the treatment of MEs(PS-GLP) group exhibited an obvious tumor inhibition in comparison with the saline and MEs-GLP-Mixture group, which could be explained by an improved tumorous accumulation based on the nanoparticle-mediated EPR effect. Furthermore, no noticeable difference in tumor inhibition between the Kanglaite^®^ injection and MEs(PS-GLP) was found, indicating good antitumor efficacy and promising prospects for clinic application. At Day 28 post administration, the mice in each group were sacrificed and tumor tissues were extracted and weighed. As shown in Figure 5(c), the results were consistent with the consequences above. In addition, the body weight did not change obviously during the treatment with each group (Figure 5(b,d)), suggesting the low cytotoxicity of the microemulsions. To further assess the toxicity of various preparations in immune organs, the weight to body ratio of liver and spleen was evaluated. The results shown in Figure 5(e) demonstrate that all microemulsion groups containing GLP caused significant liver weight increased compared with saline group. Although there is no significant difference in anti-tumor efficacy between microemulsions with or without GLP, the MEs(PS-GLP) has certain advantages in liver protection, which may be due to the hepatoprotective effects and immune enhancement function of polysaccharides extracted from *Ganoderma lucidum* (Li et al., 2015; Liu et al., 2015). For example, Li et al. found that GLP could eliminate regulatory T cell suppression of effector T cell proliferation with an increase in IL-2 secretion in hepatoma-bearing mice (Li et al., 2015). In addition, there was no significant difference in spleen indices after treatment in each group (Figure 5(f)). Above dates suggest that MEs(PS-GLP) might be efficacy and safety as an oral anticancer drug delivery system *in vivo.*

## 4. Conclusions

In this study, we developed multicomponent microemulsions combining the advantages of coix seed oil and GLP for oral delivery to lung cancer treatment. The MEs(PS-GLP) was systematically optimized by the pseudoternary phase diagram. Owing to the special carbohydrate structure of polysaccharides, the GLP was used to partly replace the surfactant used in the preparation of coix seed oil microemulsion. The MEs(PS-GLP) displayed the small particle size, homogeneous morphology and good stability under different physiological environments. Importantly, the stable binding of GLP and microemulsions was confirmed by gel-permeation chromatograph and the FRET experiment. In the A549 cells and xenograft tumors mice, MEs(PS-GLP) exhibited the obvious cytotoxicity, tumor localization, and tumor growth inhibition. This study provides a strategy for improving tumor accumulation and reducing excipient toxicity in the development of microemulsion using bioactive long chain GLP as a surfactant.

## Supplementary Material

Supporting_Information.docx
