# Supplementary material for: Ganoderma lucidum-derived polysaccharide enhances coix oil-based microemulsion on stability and lung cancer-targeted therapy
Source: Drug Deliv. 2018 Oct 20;25(1):1802–10. doi: 10.1080/10717544.2018.1516006 (PMC6201799; doi:10.1080/10717544.2018.1516006)
Supplement: Supporting_Information.docx [file IDRD_A_1516006_SM7393.docx]

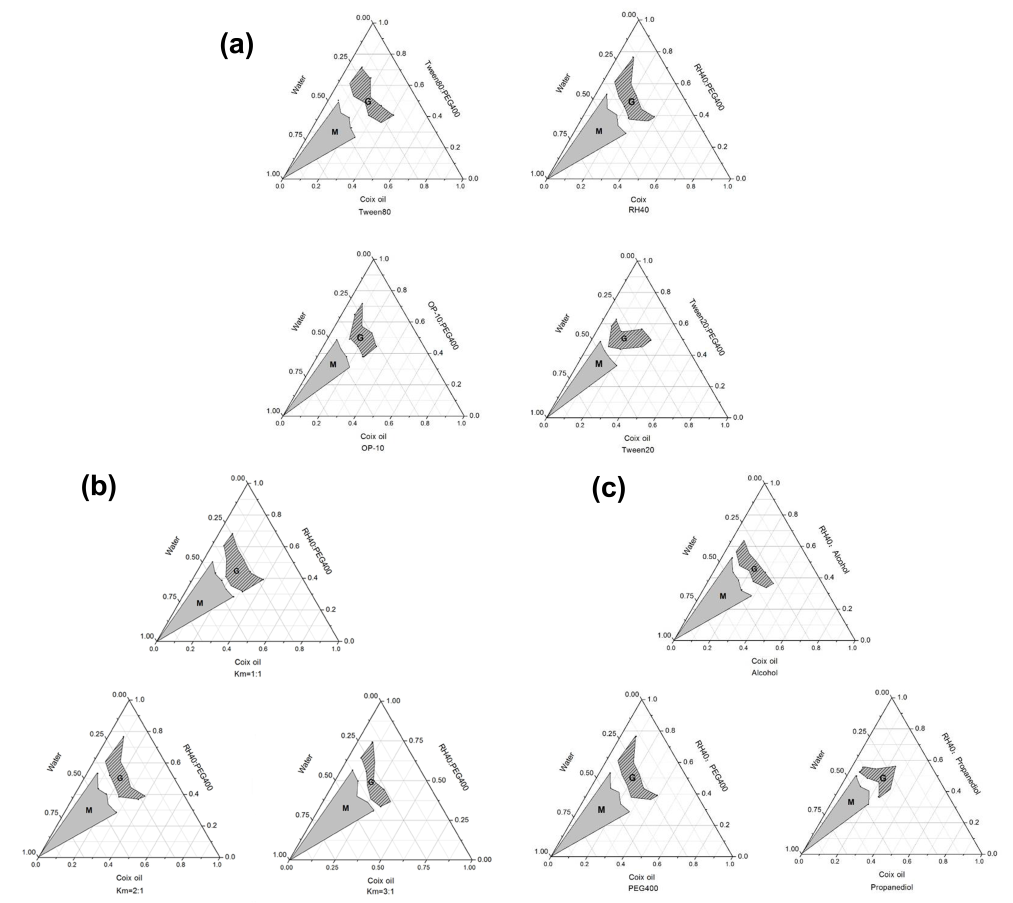


Figure S1. Pseudoternary phase diagram of microemulsion composed of different (a) surfactant, (b) Km values and (c) cosurfactant. G and M represent the zones of gel, and microemulsion, respectively.


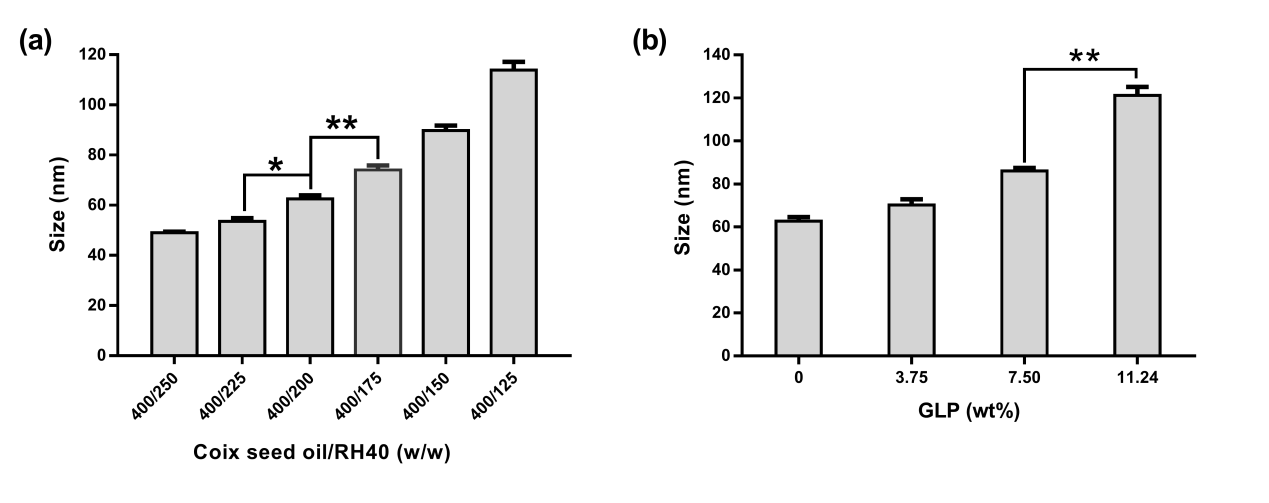


Figure S2. (a) The sizes of microemulsions with various amounts of RH40. (b) The sizes of microemulsions with various weight ratio (wt%) of GLP for equivalent surfactant RH40. **P* < 0.05, ***P* < 0.01. All the data are presented as mean ± SD (n = 3).


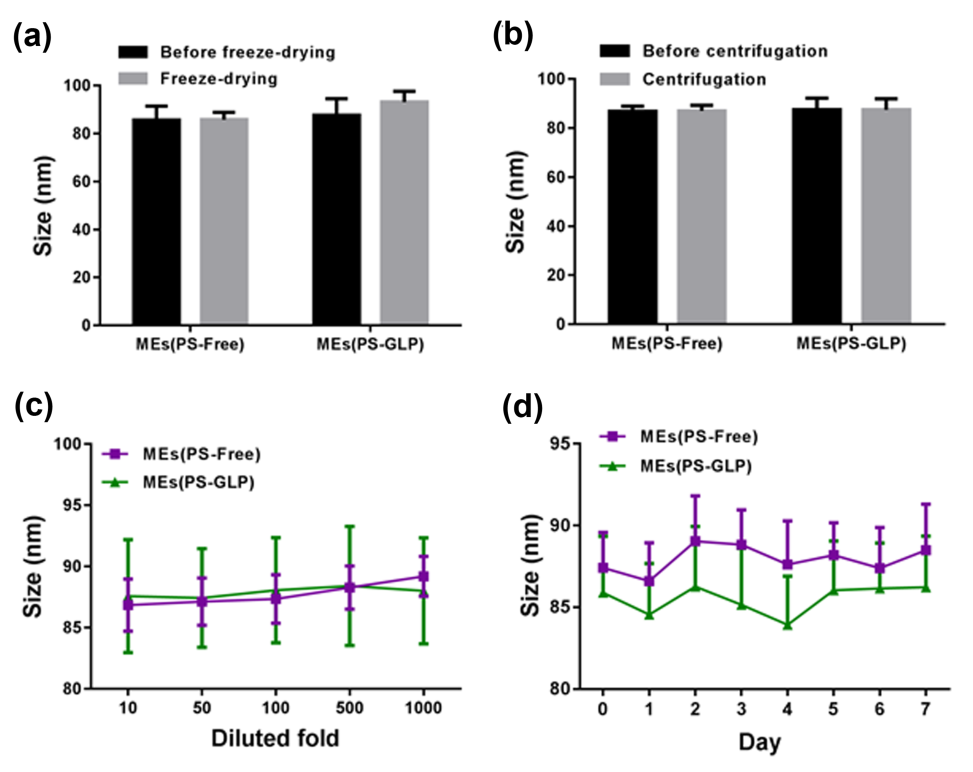


Figure S3. Stability studies of of MEs(PS-Free) and MEs(PS-GLP) after (a) freeze-drying, (b) centrifugation, (c) dilution and (d) thermocycling. All the data are presented as mean ± SD (n = 3).


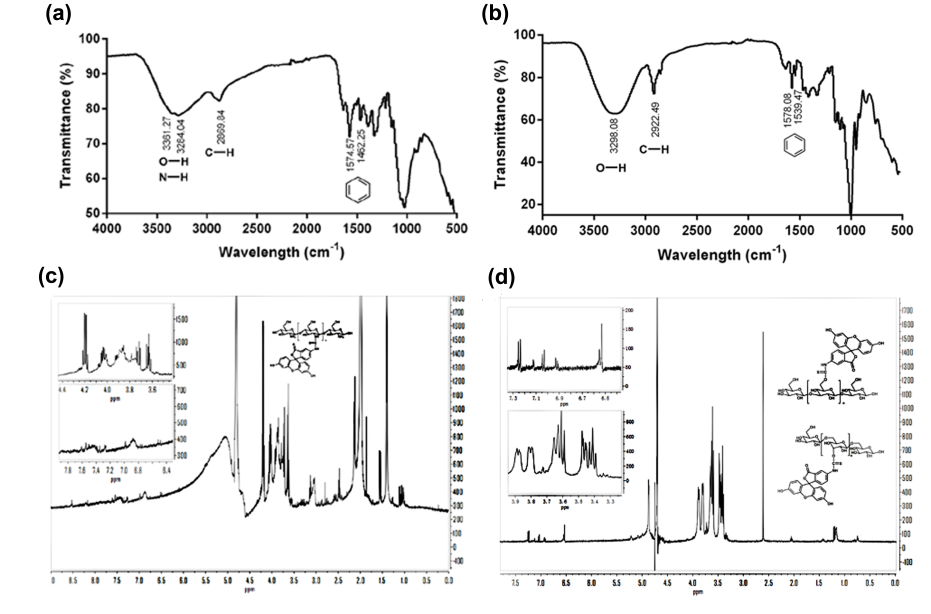


Figure S4. Characterization of CSF and DTF. (a) FTIR spectra of CSF, (b) FTIR spectra of DTF, (c) 1H NMR spectra of CSF, (d) 1H NMR spectra of DTF.





Figure S5. UV-Vis absorption of CSF, DTF and MEs.


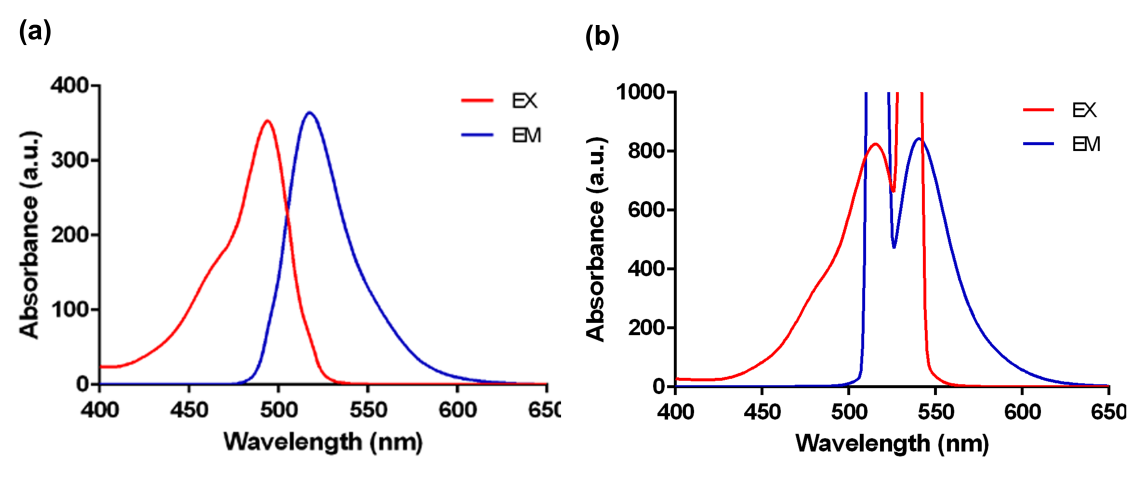


Figure S6. Fluorescent spectra of (a) CSF and (b) Rh123 MEs.


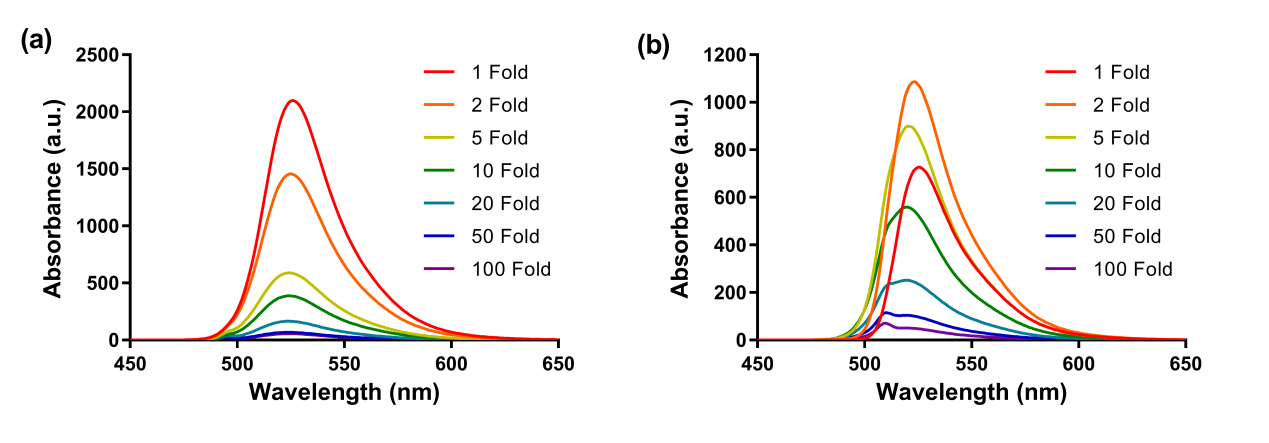


Figure S7. FRET analysis of fluorescence emission spectra of (a) the mixture of CSF and Rh123, (b) the mixture of DTF and Rh123.


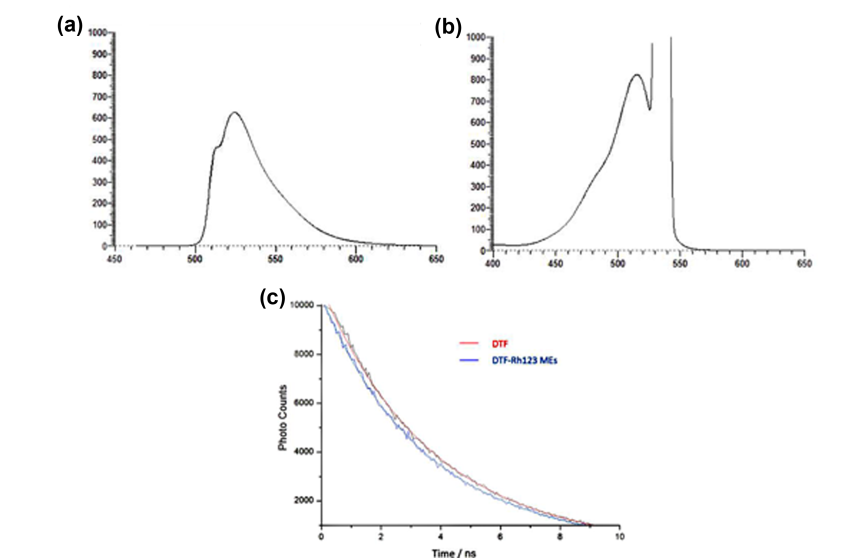


Figure S8. (a) Fluorescence emission spectra of donor of DTF. (b) The excitation spectrum of the acceptor of Rh123 MEs. (c) Fluorescence lifetime of DTF and DTF-Rh123 MEs.





Figure S9. Cell viabilities of Caco-2 cells incubated with MEs (1944CS), MEs (PS-free) and MEs (PS-GLP) at various oil phase concentrations for 48h. All the data are presented as mean ± SD (n = 6).
